# Eyeing DNA barcoding for species identification of fish larvae

**DOI:** 10.1111/jfb.15920

**Published:** 2024-09-03

**Authors:** Wan Wen Rochelle Chan, Jia Jin Marc Chang, Charles Zhiming Tan, Jie Xin Ng, Matthew Hui‐Chieh Ng, Zeehan Jaafar, Danwei Huang

**Affiliations:** ^1^ Department of Biological Sciences National University of Singapore Singapore; ^2^ Lee Kong Chian Natural History Museum National University of Singapore Singapore; ^3^ Tropical Marine Science Institute, National University of Singapore Singapore; ^4^ Centre for Nature‐based Climate Solutions National University of Singapore Singapore

**Keywords:** coral reef fish, cytochrome c oxidase subunit I, fish ecology, MinION, nanopore sequencing, reverse workflow

## Abstract

Identification of fish larvae based on morphology is typically limited to higher taxonomic ranks (e.g., family or order), as larvae possess few morphological diagnostic characters for precise discrimination to species. When many samples are presented at any one time, the use of morphology to identify such specimens can be laborious and time‐consuming. Using a reverse workflow for specimen sorting and identification leveraging high‐throughput DNA sequencing, thousands of fish larvae can be DNA barcoded and sorted into molecular operational taxonomic units (mOTUs) in a single sequencing run with the nanopore sequencing technology (e.g., MinION). This process reduces the time and financial costs of morphology‐based sorting and instead deploys experienced taxonomists for species taxonomic work where they are needed most. In this study, a total of 3022 fish larval specimens from plankton tows across four sites in Singapore were collected and sorted based on this workflow. Eye tissue from individual samples was used for DNA extraction and sequencing of cytochrome c oxidase subunit I. We generated a total of 2746 barcodes after quality filtering (90.9% barcoding success), identified 2067 DNA barcodes (75.3% identification success), and delimited 256 mOTUs (146 genera, 52 families). Our analyses identified specific challenges to species assignment, such as the potential misidentification of publicly available sequences used as reference barcodes. We highlighted how the conservative application and comparison of a local sequence database can help resolve identification conflicts. Overall, this proposed approach enables and expedites taxonomic identification of fish larvae, contributing to the enhancement of reference barcode databases and potentially better understanding of fish connectivity.

## 1 INTRODUCTION

The oceans span a large surface area at 354 million km^2^ (Costello et al., 2010) and are home to more than 35,000 marine fish species exhibiting high morphological diversity (Eschmeyer et al., 2010; Fricke et al., 2024; Hixon & Bowen, 2023). Critically, fishes undergo phenotypical metamorphosis from egg, to larva, and to adult, with significant morphological changes from the point of hatching to juvenile stages.

**FIGURE 1:**
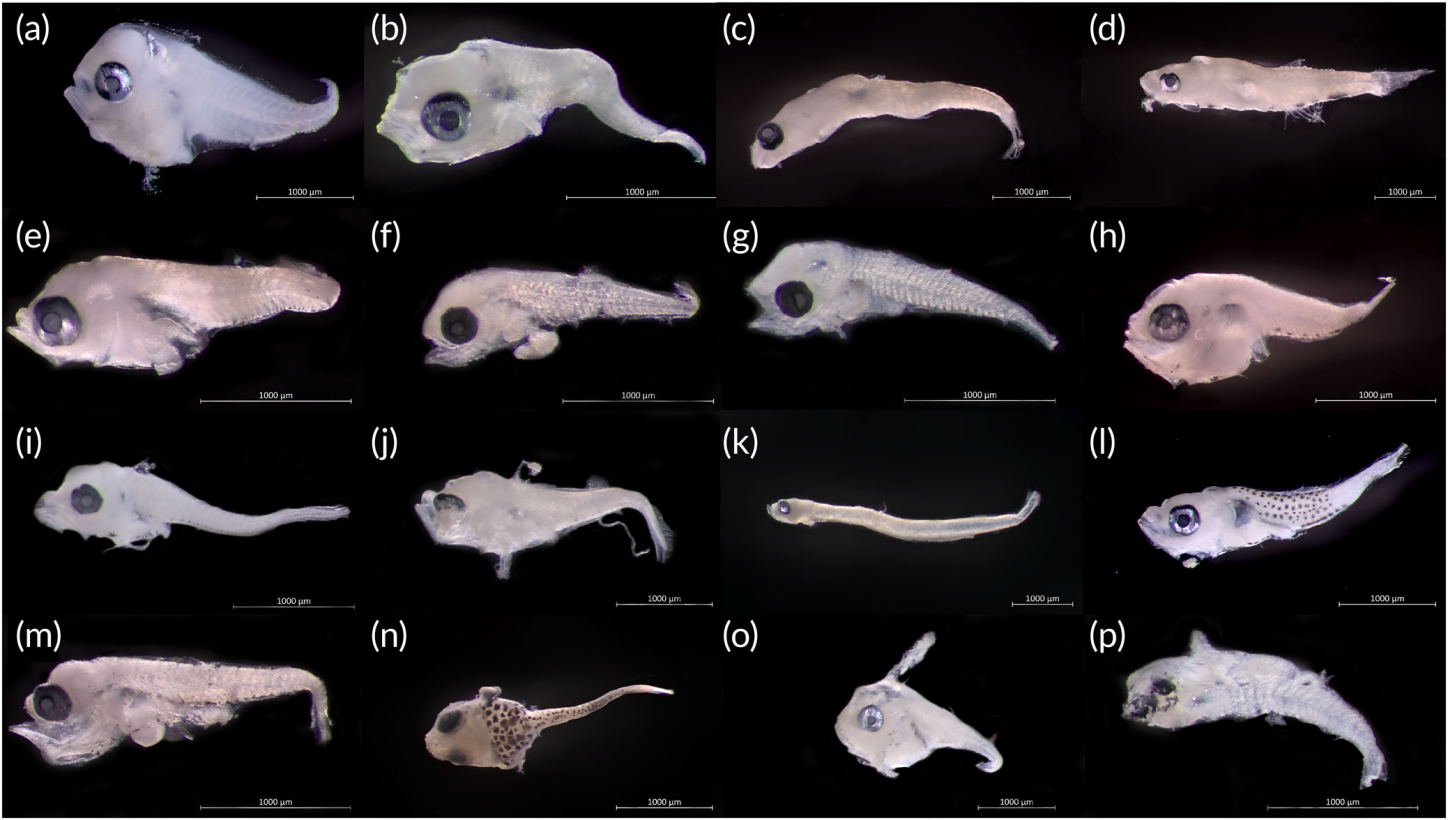
Representative images of major or important ichthyoplankton sampled. (a) Most abundant species *Leiognathus ruconius*, FISH2264, Cluster5108, and another representative species from Leiognathidae: (b) *Photolateralis stercorarius*, FISH2276, Cluster4851; most abundant family, Gobiidae: (c) *Istigobius* sp1, FISH2253, Cluster5015, and (d) *Tridentiger barbatus*, FISH2266, Cluster4921; food fishes of Nemipteridae: (e) *Scolopsis vosmeri*, FISH2243, Cluster4892, (f) *Nemipterus furcosu*s, FISH2239, Cluster4446; (g) *Pomadays maculatus*, FISH2234, Cluster5196; (h) *Pennahia anea*, FISH2273, Cluster5232; Lutjanidae: (i) *Lutjanus carponotatus*, FISH2267, Cluster4902; (j) *Lutjanus vitta*, FISH2255, Cluster5055; (k) *Encrasicholina pseudoheteroloba*, FISH2242, Cluster5114; (l) *Ostorhinchus* sp., FISH2281, Cluster4983; (m) *Atule mate*, FISH2263, Cluster5157; (n) *Pegasus volitans*, FISH2238, Singleton7; (o) *Monacanthus chinensis*, FISH2278, Cluster5048; (p) Actinopterygii sp., FISH2237, Singleton118.

Marine fishes in early ontogenetic stages—often referred to as “fish larvae” or “ichthyoplankton”—are challenging to identify based on morphology due to convergent traits in development for many taxa (e.g., Gobiidae Cuvier, 1816 in Figure 1c,d and Lutjanidae Gill, 1861 in Figure 1i–k). Closely related fish species appear identical pre‐flexion and exhibit similar meristic and morphometric characters in their early life stages (Ko et al., 2013; Victor et al., 2009). Ichthyologists have traditionally used a suite of morphological characters (e.g., body shape, pigmentation, meristic count) to identify fish larvae (Ko et al., 2013), but limited diagnostic discrimination of species results in identification to higher taxonomic hierarchies, such as familial or generic levels (Hubert et al., 2010; Leis & Carson‐Ewart, 2000; Neigel et al., 2007). When specific morphological characters are not apparent or undeveloped in larval stages, the conservative approach is adopted, again resulting in identification to higher taxonomic hierarchies at the familial, rather than the generic or specific, levels (Azmir et al., 2017; Ko et al., 2013). Further, morphological characters may be insufficient to distinguish rare and cryptic species at the larval stages (Ko et al., 2013). The interpretation of morphological characteristics also contributes to uncertainties in identification accuracy as biases persist based on expertise of the identifier (Krell, 2004). If only morphological characters were solely used for identification of fish larvae, studies on biodiversity and community structure could fail to reflect accurate community changes (Ko et al., 2013) and be subject to biases toward fish clades and life stages that are identified more easily (Mikkelsen & Cracraft, 2001; Neigel et al., 2007).

The importance of DNA sequencing in complementing identification of fishlarvae is widely acknowledged (Azmir et al., 2017; Hulley et al., 2018; Lira et al., 2023). Through initiatives such as FISH‐BOL (Fish Barcode of Life, http://www.fishbol.org, the barcode of mtDNA cytochrome c oxidase subunit I [COI] sequences), which started in 2004, more than 435,400 COI barcodes, belonging to at least 25,000 marine and freshwater fish species (accessed: May 20, 2024), have been generated (Ko et al., 2013; Victor, 2015; Ward et al., 2009). A study conducted in the La Reunion exclusive economic zone (EEZ) is an example of a fish larval DNA barcoding campaign that has improved overall fish biodiversity knowledge (i.e., recording of 55 new Barcode Index Numbers [BINs] and the addition of new species to an updated checklist) (Collet et al., 2018). The molecular approach has also been recognized as the most definitive method to accurately identify fish eggs to the species level (Shao et al., 2002), with the caveat that identifications of sequences within the resource library are correct. Projects such as FISH‐BOL have also revealed numerous fish lineages with distinct COI sequences yet possessing similar morphologies (i.e., cryptic species) (Victor, 2015), adding to the challenges of solely using morphological traits in taxonomic identification.

Relatedly, genetic methods for ichthyoplankton identification (primarily DNA metabarcoding) have been applied widely (Djurhuus et al., 2018; Schroeder et al., 2020). DNA metabarcoding is a simple, effective, and popular method that allows for the rapid detection of ichthyoplankton (e.g., 12,220 species of Actinopterygii) recorded on MetaZooGene Barcode Atlas and Database (MZGdb; https://metazoogene.org/MZGdb; accessed: December 18, 2023) (Bucklin et al., 2021; Taberlet et al., 2012); however, this method fares poorly when assessing the relative proportion of each taxon within a sample (Taberlet et al., 2012). Therefore, DNA barcoding remains critical for biodiversity research as individual samples can be morphologically verified, and species abundance and richness can be estimated.

The use of several techniques in gene sequencing can aid the process of DNA barcoding. Specifically, the use of the HotSHOT DNA extraction (Truett et al., 2000) generates PCR‐ready extracts immediately after extraction, significantly shortening the workflow by a DNA purification step (Lunt, 2017; Truett et al., 2000). The naturally enriched mitochondrial genome, and thus COI, typically constitutes 0.5%–5% of the DNA in a genomic extraction and would be of sufficient quality even without purification (Crampton‐Platt et al., 2016). This method has been proven to be reliable, inexpensive, straightforward, and time‐efficient for simple DNA extraction in individual tubes. Only a small amount of tissue is required, and chances for cross‐contamination are reduced (Montero‐Pau et al., 2008; Srivathsan et al., 2021; Zieritz et al., 2018). Furthermore, DNA barcodes can now be generated in a high‐throughput, time‐efficient, and cost‐effective way with nanopore sequencing. Next‐generation sequencing (NGS) on platforms like Illumina is already advantageous over Sanger sequencing, where the sequencing of many pooled samples can greatly lower the cost per barcode (Meier et al., 2016). Nanopore additionally allows sequencing on small and portable MinION sequencers with minimal, basic laboratory equipment, enabling the sequencing of many specimens even in the field under a wide range of environmental conditions (Chang et al., 2020, 2020; Chang et al., 2022; Srivathsan et al., 2021). Importantly, MinION sequencers allow users to retrieve data near‐instantaneously upon commencing a sequencing run (Srivathsan et al., 2023). Although these sequencers have generated sequences with error rates of 10%–15% (Wick et al., 2019), the consensus DNA barcode error rates are now comparable to other high‐throughput sequencing platforms for more recent sequencing chemistries (Srivathsan et al., 2021; Vierstraete & Braeckman, 2022).

Barcoding for fishes has been based on a few mtDNA markers including COI, cytochrome b, 12S, and 16S. More focus has been placed on using COI, following the primers designed by several key groups of researchers (e.g., Hebert et al., 2003; Ward et al., 2005). Ivanova et al. (2007) later proposed the use of universal primer cocktails for fish DNA barcoding to simplify the four pair‐wise permutations of the primers proposed by Ward et al. (2005). The COI‐3 primer cocktail was designed for fishes and has been proven to be effective (Ho et al., 2020; Moran et al., 2016).

In this study, we amplified the mitochondrial COI locus (652 bp) for processing thousands of fish larvae samples and sorting them into putative species with DNA barcodes. This is coined by Wang et al. (2018) as the “reverse workflow,” as traditionally, all specimens from bulk sampling would be morphologically sorted, with a select few specimens verified using DNA barcoding as the latter was more costly. With reductions in sequencing costs, we are now able to sequence all the samples, sort them based on similarities in DNA sequences, and follow up with morphological work (if needed) on these sorted species units. Using time‐efficient sequencing methodology using HotSHOT DNA extraction and MinION sequencer, all fish larvae specimens can be barcoded and efficiently sorted into genetic clusters. The number of clusters required for assessment of morphological species validation is thus reduced, immensely shortening the workflow (Hartop et al., 2022; Wang et al., 2018). Our approach paves the way for more efficient DNA barcoding pipelines for fish larvae and assists in processing these specimen‐rich samples.

## 2 MATERIALS AND METHODS

### 2.1 Specimen collection

**FIGURE 2:**
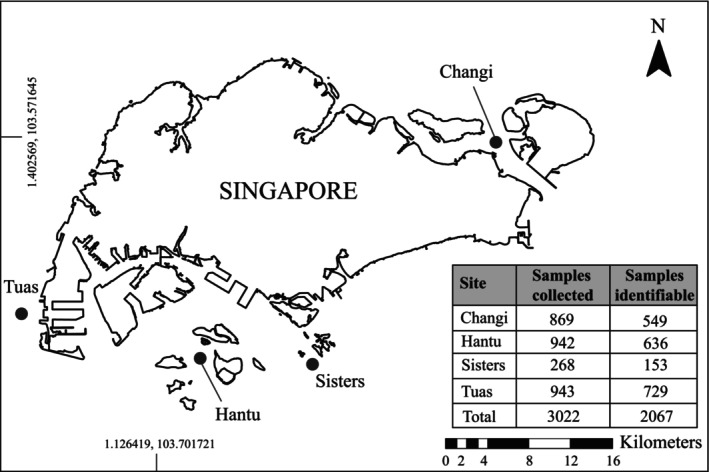
Location of sites sampled in Singapore where plankton tows were conducted to collect fish larvae. Base map was adapted from ArcGIS. Insert table displays the count of samples collected using plankton tows and samples which were identified, sorted by site.

We conducted plankton tows for fish larvae at four sites within the territorial waters of Singapore—off Changi Beach (Changi), off Pulau Hantu (Hantu), off Sisters' Islands Marine Park (Sisters), and off Tuas Coast (Tuas)—in February and March 2022 (Figure 2). Hantu and Sisters are located within the Straits of Singapore, adjacent to coral reefs habitats. Changi and Tuas are situated within the Straits of Johor, adjacent to soft‐substrate habitats, such as seagrass meadows and mudflats. Both Changi and Tuas could be important connectivity channels, linking the waters of Singapore to key water bodies—South China Sea for Changi and Indian Ocean through the Malacca Strait for Tuas (Tay et al., 2016).

A bongo net (diameter 50 cm, mesh‐size 500 μm) was deployed from a moving boat to an approximate depth of 15 m and towed obliquely for 15 min. Three plankton tow replicates were conducted per site. Specimens were preserved in situ and in bulk in ≥70% molecular‐grade ethanol. The specimens were later sorted under a dissecting microscope (Nikon SMZ745) to select and preserve each ichthyoplankton in individual tubes with 100% molecular‐grade ethanol for long‐term storage. We selected a handful of specimens for photography to demonstrate the feasibility of the reverse workflow.

### 2.2 Ethics statement

All applicable national and/or institutional animal welfare laws and guidelines were followed, as approved by the National Parks Board research and collection permit (NP/RP22‐022) and Institutional Animal Care and Use Committee (IACUC) (protocol number: B21‐1266).

### 2.3 DNA extraction

Tissue from all 3022 samples was extracted with a clean needle under a dissecting microscope (Leica M205C). To ensure minimal damage to the specimen, the right eye (< 1 mm in diameter) was used for DNA extraction (Nonaka et al., 2021). The left side of fishes is conventionally used in photography of scientific specimens—positioned with the horizontal body axis and left side facing up (Motomura et al., 2013). For samples without the right eye or of low quality, body tissue of an equivalent size to the eye was dissected instead. We additionally extracted tissue from larvae from other sampling occasions as positive controls.

Each dissected sample was placed in individual wells of 96‐well plates, and genomic DNA was extracted using the HotSHOT method (Truett et al., 2000) according to the protocol by Lunt (2017). Tissue samples were immersed in 25 μL of alkaline lysis solution, incubated at 95°C for 30 min in a thermocycler, and an equal volume (25 μL) of neutralizing solution was subsequently added. These were thereafter maintained at 2 or −20°C for extended storage.

### 2.4 COI amplification

The 652‐bp region of the mitochondrial COI was amplified using PCR with the COI‐3 universal fish primer cocktail, which includes two primer pairs (Ivanova et al., 2007). All four primers were used for amplification in each PCR. The first primer pair was FishF2_t1: 5′‐TCG ACT AAT CAT AAA GAT ATC GGC AC‐3′ and FishR2_t1: 5′‐ACT TCA GGG TGA CCG AAG AAT CAG AA‐3′. The second primer pair was VF2_t1: 5′‐TCA ACC AAC CAC AAA GAC ATT GGC AC‐3′ and FR1d_t1: 5′‐CAC CTC AGG GTG TCC GAA RAA YCA RAA‐3′. To ensure samples could be pooled for sequencing and individually identified downstream, the 5′‐end of each primer was uniquely tagged with custom 13‐bp tag sequences, where each sample had a unique forward and reverse tag combination (Meier et al., 2016; Srivathsan et al., 2019; Srivathsan & Meier, 2024) (Supporting information file 1). More details regarding the workflow for the amplification can be found in Supporting information file 2.

For each PCR reaction, 2 μL of HotSHOT extracted DNA, 1 μL each of 10‐μM 13‐bp tagged primer, 2 μL of bovine serum albumin (1 mg/mL), and 12.5 μL of GoTaq Green Master Mix (Promega) were topped up to 25 μL with nuclease‐free water. The thermocycle profile was as follows: 5 min initial denaturation at 94°C, followed by 35 cycles of denaturation at 94°C (1 min), 47°C (1 min), 72°C (1 min), with a final extension at 72°C (5 min). PCR amplification of all samples and negative controls (PCR and extraction controls) were visualized on 2% agarose gels. PCR products were then pooled in equal volumes and cleaned using 0.6× ratio of AMPure XP beads (Beckman Coulter). We prepared two libraries using the Ligation Sequencing Kit (SQK‐LSK114) according to manufacturer's recommendations.

### 2.5 MinION barcoding and bioinformatics

The libraries were sequenced on a MinION sequencer (Oxford Nanopore Technologies [ONT]) in two runs; Run 1: 24 h on *MinKNOW* v23.04.5 and Run 2: 72 h on *MinKNOW* v23.04.6. The first run was conducted on a fresh R10.4.1 (FLO‐MIN114) flow cell, whereas the second was conducted on the same flow cell but washed prior according to manufacturer instructions. We ensured that different tag combinations were used to prevent any cross‐contamination between runs. Sequences were then screened for any previous tags prior to downstream analysis. Basecalling of pod5 reads was performed on computer servers using *dorado* v.0.3.0 (model: dna_r10.4.1_e8.2_400bps_sup@v4.2.0, min *Q*‐score: 12) and converted to fastq format (Kuśmirek, 2023). *ONTbarcoder* v0.1.9 (Srivathsan et al., 2021) was used to demultiplex and generate consensus barcodes for both runs and both primer pairs. Default settings were used, with the only changes being the minimum barcode length (set to 652) and the use of the “Vertebrate Mitochondrial Code” as the genetic code. *ONTbarcoder* subsampled the reads for each sample (beginning with 25×, then 50×, 100×, 200×, 500×) to build a consensus sequence by length. For samples that failed to yield a consensus, *ONTbarcoder* attempted to build a consensus by similarity; the selected coverage used for alignment building, most similar to the preliminary barcode, was 100. The “QC_compliant” output file for both primer sets was compared using the “Compare barcode sets” function on *ONTbarcoder*. “QC_compliant” barcodes are consensus that are translatable, match the specified barcode length (652 bp), free of ambiguous “N” bases, and do not cause insertion of gaps when aligned. “QC_compliant” barcodes are considered the “highest‐quality” barcodes and satisfy the quality checks discussed above, without any corrections (Srivathsan et al., 2021). Identical barcodes from both primer pairs were merged. Samples with different DNA barcodes (fewer than 10‐bp difference and close to 100% alignment) for both primer sets were put through the *BLAST* web server to ensure that the differences did not result in a different identity match. These were then consolidated with the merged identical barcodes. The consolidated dataset was filtered to retain barcodes with more than 10× read coverage and then aligned using *MAFFT* v7.453 (Katoh & Standley, 2013). *Objective clustering* (Meier et al., 2006) was used to group sequences based on *p*‐distances (Mabragana et al., 2011) into molecular operational taxonomic units (mOTUs), which were uniquely numbered as “Cluster” or “Singleton.” We clustered sequences at 2%, 3%, and 4% *p*‐distances to evaluate the sensitivity of the mOTUs to different distance thresholds (Ip et al., 2021; Wang et al., 2018).

The mOTUs were matched using blastn function in *BLAST+* v2.13.0 (Camacho et al., 2009) to a NCBI GenBank *nt* database (downloaded July 14, 2023; e‐value: 10^−6^ and percentage identity ≥80%). *BLAST+* matches were parsed through *readsidentifier* v1.0 (Srivathsan et al., 2015) to summarize the taxonomic hits. All mOTUs were then matched to a curated local database from a previous project (GenBank sequences to be released in 2025). The local database was used to improve *BLAST+* matches of <98% to increase the overall number of taxonomic identifications. For *BLAST+* matches with ≥98% match, we used the local database to improve these identifications to as low a level as possible (e.g., an improvement from generic to specific level) and for more updated nomenclature where appropriate. Where barcodes were matched at ≥98% by both databases to different identifications, we evaluated all hits and justified each of the accepted identifications. When uncertain, we took a conservative approach by adopting congruent identities from both databases. For example, if a sample matches to the same genus but different species for both databases, the sample would be identified by its genus name. We additionally matched mOTUs to the Barcode of Life Data System (BOLD) database with *BOLDigger* v2.1.0 (Buchner & Leese, 2020) to understand if identifications would be improved. Samples that “passed” refer to those successfully matched ≥98% to a database; we were able to attach a name to a barcoded sample, and these samples were identifiable.

### 2.6 Diversity analysis

Barcodes that matched with ≥98% to the NCBI *nt* database, with the additional use of the local database, were selected for ecological analysis. Data were sorted using *dplyr* v1.1.0 (Wickham et al., 2023), and graphical figures were plotted using *ggplot2* v3.4.1 (Wickham, 2016) and *VennDiagram* v1.7.3 (Chen & Boutros, 2011) on R v4.2.2 (R Core Team, 2022). We used the *iNEXT* (iNterpolation/EXTrapolation) v3.0.0 R package to calculate rarefaction, alpha diversity, and to construct species accumulation and sample coverage curves (Chao et al., 2014; Hsieh et al., 2016).

## 3 RESULTS

### 3.1 Specimen collection and DNA barcoding

We obtained a total of 3022 fish larval specimens across four sites in Singapore. Tuas yielded the most larvae at 943 specimens, whereas Sisters had the fewest at 268 specimens (Figure 2).

A total of 7,667,080 raw sequence reads were generated for Run 1, of which 6,515,524 were used for demultiplexing for primer pair Fish F2 and Fish R2, whereas 6,515,377 were used for demultiplexing for primer pair Fish VF2 and Fish FR1d after passing the length filter. *ONTbarcoder* successfully demultiplexed 3,916,050 reads (60.1% success) for primer pair Fish F2 and Fish R2, and 3,921,638 reads (60.2% success) for primer pair Fish VF2 and Fish FR1d (Table 1). Run 2 generated 5,441,189 raw reads, of which 4,228,396 were used for demultiplexing for primer pair Fish F2 and Fish R2, whereas 4,224,998 were used for demultiplexing for primer pair VF2 and FR1d after passing the length filters. Similarly, *ONTbarcoder* successfully demultiplexed 2,516,141 reads (59.5% success) for primer pair Fish F2 and Fish R2, and 2,526,411 reads (59.8% success) for primer pair Fish VF2 and Fish FR1d (Table 1). For both runs and primer pairs, demultiplexing successes were close to 60% (Table 1). It is important to note that the sequencing runs also included PCR products from other experiments not evaluated in this study, which contributed to the lower demultiplexing success rate. The demultiplexing success was higher than that reported in a previous study (44.8%) (Srivathsan et al., 2023) but lower than in other studies (at least 84.2%) (Cuber et al., 2023; Srivathsan et al., 2021). In any case, barcode calling was not adversely affected by the lower demultiplexing success as the lowest median sequencing depth was 787 reads per sample (Supporting information file 3).

**TABLE 1:** Number of samples and controls sequenced, reads demultiplexed (percentage demultiplexing success indicated in parentheses), and quality‐checked barcodes obtained for each run and primer pair.

Primer pairs	Run 1	Run 2
	Fish F2‐fish R2/VF2‐FR1d	Fish F2‐fish R2/VF2‐FR1d
Samples	2114	908
Positive controls	65	0
Negative controls	95	12
Raw reads	7,667,080/7,667,080	5,441,189/5,441,189
Reads retained	6,515,524/6,515,377	4,228,396/4,224,998
Demultiplexed reads	3,916,050 (60.1%)/3,921,638 (60.2%)	2,516,141 (59.5%)/2,526,411 (59.8%)
Barcodes	1946/1951	845/845

The number of compliant barcodes was similar for both primer pairs and across both runs (Table 1). Comparing results of both primer pairs within each run, we found that >99% of barcodes were identical (Run 1: 1943 barcodes and Run 2: 841 barcodes). Five pairs of incompatible barcodes (at least one base pair difference) were found (FISH2097, FISH2873, FISH3007, FISH2395, and FISH0787). These pairs of barcodes had fewer than eight base pair differences and showed no difference in taxonomic identification when compared using BLAST; therefore, they were accepted. Additionally, two barcodes were only picked up by the primer pair Fish F2 and Fish R2, and seven barcodes were only picked up by the primer pair VF2 and FR1d. Results from both runs were combined, together with the barcodes with no difference in taxonomic identifications and barcodes picked up by only one primer pair to make up the overall combined dataset prior to aligning and clustering.

When clustered at 2%, 3%, and 4% pair‐wise differences, most clusters were stable (Supporting information file 4), suggesting that 2% was a suitable threshold to delimit mOTUs (Mabragana et al., 2011). However, the shorthead anchovy (*Encrasicholina heteroloba* Rüppell, 1837), together with eight other pairs of distinct clusters, had common taxonomic identifications when clustered at 2% (Table 2). Through thorough checks of associated sequences on GenBank and within literature, we were able to distinguish Cluster5162 as *E. heteroloba* (matched 100% to GenBank accession number MT080435) and Cluster5114 as *E. pseudoheteroloba* (matched 100% to GenBank accession number MT080421) (Hata & Motumora, Hata & Motomura, 2017; Lavoué et al., 2022). Only three pairs merged when pair‐wise differences were increased to 2.8% (*Mugilogobius chulae* [Smith, 1932] and *Upeneus sulphureus* Cuvier, 1829) and 3.8% (*Parachaeturichthys polynema* [Bleeker, 1853]), whereas the other five pairs maintained as separate clusters (Table 2). For the former three pairs, merging at higher percentage differences could be due to intraspecific diversity. For those that did not merge even with increased pair‐wise differences, the different clusters could possibly represent separate species that were matched to the same taxonomic identification reported in the databases. To avoid artificially collapsing these clusters, we standardized the pair‐wise difference at 2% for the whole dataset and retained them as separate clusters for the final taxonomic identification.

**TABLE 2:** Pairs of clusters with similar or identical species matched at different pair‐wise clustering percentages.

Species matched	Percentage pair‐wise similarity	Cluster 4.5%	Cluster 3.8%	Cluster 3.3%	Cluster 2.8%	Cluster 2%
*Boleophthalmus boddarti*	88.3	Cluster5233	Cluster5233	Cluster5233	Cluster5233	Cluster5233
		Singleton2251	Singleton2251	Singleton2251	Singleton2251	Singleton2251
*Encrasicholina heteroloba*	86.6	Cluster5162	Cluster5162	Cluster5162	Cluster5162	Cluster5162
		Cluster5114	Cluster5114	Cluster5114	Cluster5114	Cluster5114
*Leiognathus ruconius*	91.9	Cluster5108	Cluster5108	Cluster5108	Cluster5108	Cluster5108
		Cluster2823	Cluster2823	Cluster2823	Cluster2823	Cluster2823
*Mahidolia mystacina*	83.5	Cluster4772	Cluster4772	Cluster4772	Cluster4772	Cluster4772
		Singleton1084	Singleton1084	Singleton1084	Singleton1084	Singleton1084
*Mugilogobius chulae*	97.1	**Cluster5257**	**Cluster5257**	**Cluster5257**	**Cluster5257**	Cluster4567
		**Cluster5257**	**Cluster5257**	**Cluster5257**	**Cluster5257**	Singleton1660
*Nectamia savayensis*	88.8	Cluster4950	Cluster4950	Cluster4950	Cluster4950	Cluster4950
		Singleton163	Singleton163	Singleton163	Singleton163	Singleton163
*Parachaeturichthys polynema*	96.2	**Cluster5260**	**Cluster5260**	Cluster5094	Cluster5094	Cluster5094
		**Cluster5260**	**Cluster5260**	Cluster5030	Cluster5030	Cluster5030
*Sunagocia carbunculus*	85.3	Cluster4951	Cluster4951	Cluster4951	Cluster4951	Cluster4951
		Cluster4558	Cluster4558	Cluster4558	Cluster4558	Cluster4558
*Upeneus sulphureus*	97.2	**Cluster5256**	**Cluster5256**	**Cluster5256**	**Cluster5256**	Cluster4967
		**Cluster5256**	**Cluster5256**	**Cluster5256**	**Cluster5256**	Singleton320

*Note*: Merged clusters at higher thresholds are indicated in bold.

A total of 343 mOTUs were obtained (Supporting information file 5) when clustered at 2%. Of the 3087 samples (including 65 positive controls; Supporting information file 1) sequenced, 2792 MinION barcodes were generated. After contaminant barcodes and positive controls were removed (Supporting information file 6), 2746 fish barcodes (90.9% barcoding success) and 341 mOTUs remained (Supporting information file 5). This was higher than the PCR success rate estimated by agarose gel electrophoresis for successful samples (80.9%).

Preliminary analyses included the matching of sequences to BOLD database (Supporting information file 5). However, we found two main challenges with using the BOLD database for classification: (1) many sequences were not published (i.e., from “private” collections) where details of the collection were not accessible for verification; (2) many sequences were mined from NCBI *nt* database. We eventually focused on using the NCBI *nt* database (which comprises information uploaded on BOLD database and can be verified) and the local database (which was based on verifiable voucher specimens) for the identification of mOTUs in this study. Results are based on matches to the NCBI *nt* and local sequence databases only.

**FIGURE 3:**
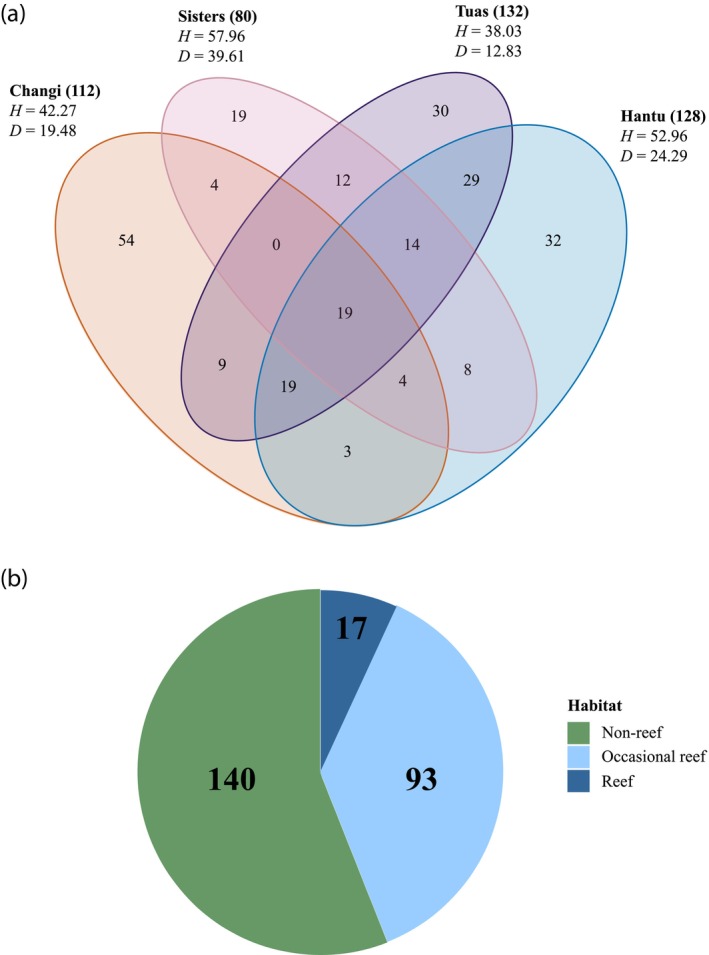
(a) Breakdown of molecular operational taxonomic units (mOTUs) across study sites. Total mOTU count at each site in parentheses. Shannon diversity (H) and Simpson diversity (D) are also included per site. (b) Typical habitat type information (highly reef‐associated, occasional reefs, non‐reef) for the adults of each matched mOTU. We were unfortunately unable to distinguish the habitat preference for mOTUs identified higher than species level.

With the combined use of NCBI *nt* database and local sequence database, we identified a total of 2067 confirmed DNA barcodes for one sampling month (75.3% identification success) that constituted 256 mOTUs (Figure 3a; Supporting information files 4 and 6). All COI sequences have been submitted to NCBI GenBank (accession numbers: PP088104–PP090879).

Of 256 mOTUs that were identifiable, the *nt* database alone was able to match 215 barcodes (84.0% mOTU identification success), and the local sequence database alone successfully matched 179 barcodes (69.9% mOTU identification success). The local sequence database identified seven barcodes not found in the *nt* database, whereas the *nt* database identified only one barcode not found in the local database (Supporting information file 5). The *nt* database additionally improved the identification of 19 barcodes, whereas the local sequence database refined the identification of 12 barcodes to a higher taxonomic level (Supporting information file 5). Nevertheless, the use of two databases resulted in 18 conflicting identities (Table 3). Most conflicts differed only at the species level and two had identifications of different genera (Cluster3325 and Cluster5100). Cluster5177 had originally matched identifications from different orders for which we undertook thorough investigative work to accept only the most accurate species identification. We elaborated on the justifications for the final accepted identifications in Table 3.

**TABLE 3:** Clusters with conflicting identities, when matched ≥98% to distinct species to NCBI *nt* database and the local database.

Cluster identification	NCBI identification (%)	Local database identification (%)	Notes	Final identification
Cluster3325	*Photopectoralis bindus* (100)	*Nuchequula* sp1 (100)	Both genera are valid	Leiognathidae sp.
Cluster5100	*Paramonacanthus* sp. (100)	*Monacanthus* sp1 (100)	Both genera are valid	Monacanthidae sp.
Cluster3020	*Lepidamia kalosoma* (99.2)	*Apogonichthyoides taeniatus* (100)	Both genera are valid	Apogonidae sp.
Cluster3041	*Eubleekeria jonesi* (100)	*Leiognathus equula* (100)	Both species are valid	Leiognathidae sp.
Cluster3107	Apogon sp. (100)	*Ostorhinchus cavitensis* (100)	Based on photograph of matched GenBank entry (MK777379)	*O. cavitensis*
Cluster3745	*Sphyraena putnamae* (100)	*Sphyraena jello* (100)	Most matched ≥98% to *S. jello*. Only one matched to *S. putnamae* (KP856819), likely as a single misidentified specimen.	*S. jello*
Cluster4477	*Ambassis nalua* (100)	*Ambassis kopsii* (100)	Both species are valid	*Ambassis* sp.
Cluster4491	*Deveximentum indicium* (99.8)	*Deveximentum hanedai* (99.8)	Both species are valid	*Deveximentum* sp.
Cluster4624	*Ambassis dussumieri* (99.8)	*Ambassis vachellii* (99.8)	Both species are valid	*Ambassis* sp.
Cluster4631	*Scolopsis vosmeri* (100)	*Scolopsis japonicus* (100)	Taxonomy for the *S. vosmer*i species complex was recently clarified (Russell et al., 2022). Cluster4631 matched at 100% to an entry (KY315395), which Russell et al. (2022) had revised as *S. japonicus*.	*S. japonicus*
Cluster5007	*Alepes vari* (100)	*Alepes djedaba* (100)	Both species are valid	*Alepes* sp.
Cluster5156	*Amblyeleotris macronema* (99.343)	*Amblyeleotris gymnocephala* (99.7)	Based on photograph of matched GenBank entry (MK777316)	*A. gymnocephala*
Cluster5191	*Ambassis vachellii* (99.8)	*Ambassis interrupta* (99.8)	Both species are valid	*Ambassis* sp.
Cluster5216	*Taeniamia kagoshimanus* (100)	*Taeniamia fucata* (99.8)	Both species are valid	*Taeniamia* sp.
Cluster5221	*Cryptocentrus leptocephalus* (100)	*Cryptocentrus melanopus* (100)	Taxonomy clarified in Hoese et al. (2011)	*C. melanopus*
Singleton1465	*Cryptocentrus cebuanus* (98.8)	*Cryptocentrus pavoninoides* (99.1)	Both species are valid	*Cryptocentrus* sp.
Cluster5208	*Pomadasys hasta* (99.8)	*Pomadasys kaakan* (99.8)	*P. hasta* is now accepted as *Pomadasys argenteus*. Both *P. argenteus* and *P. kaakan* are valid.	*Pomadsys* sp.
Cluster5177	*Sparus aurata* (99.8)	*Omobranchus ferox* (99.4)	*S. aurata* likely stemmed from one misidentification that propagated to other GenBank entries (e.g., OK012057, OQ857169, and KX223953) and even affecting published papers (Azmir et al., 2020; Jiang et al., 2021; Nuryanto et al., 2023). When validated against whole mitochondrial *S. aurata* sequences (KT805959, NC_024236, and OD909577), Cluster5177 matched 84.6% at best, suggesting that species identification *S. aurata* is inaccurate. Cluster5177 matched more accurately to *O. ferox* at 99.4% identity (MG210400), in which Gibbs et al. (2018) performed phylogenetic analyses of the clade *Omobranchus* using cytochrome c oxidase subunit I (COI) and four nuclear genes. This identification is further supported by the curated local database.	*O. ferox*

*Notes*: Percentage identity matches in parentheses. The main approach used to resolve conflicting matches was to accept the lowest taxonomic level encompassing both identifications. Reasons differing from this were elaborated under the “Notes” column with related GenBank accession numbers stated in parentheses where appropriate.

### 3.2 Diversity analysis

**FIGURE 4:**
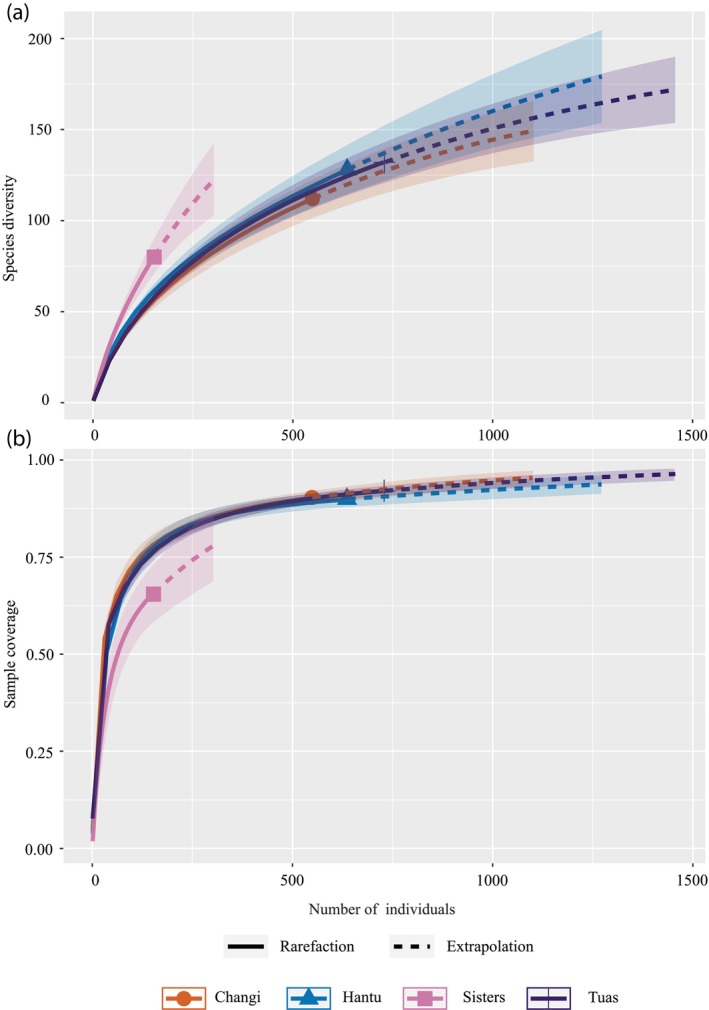
(a) Species accumulation curve with overlapping confidence intervals for all sites except Sisters Island. (b) Sample coverage curve approaching the plateau with overlapping confidence intervals for all sites except Sisters Island.

Overall, 256 mOTUs (i.e., 256 species from 52 families) were identified, which was proportional to the number of specimens collected per site (Table 4; Figure 1). Specimens collected off Tuas had the highest number of identifiable samples and the greatest number of mOTUs (Figure 3a). Of these, 19 mOTUs were shared across all four sites, and the site with the greatest number of unique mOTUs was Changi (Figure 3a). Despite having the lowest numbers and least number of mOTUs belonging solely to this site, Sisters appeared to host the highest species diversity (Figures 3a and 4a), but additional sampling would be required for more precise diversity estimates. With more than 500 samples each, the sampling efforts for Changi, Sisters, and Tuas appeared to be nearing completeness (Figure 4b).

**TABLE 4:** Sample abundance of top 20 clusters and final identification per site.

Cluster identification	Family	Final identification	Changi	Hantu	Sisters	Tuas	Total
Cluster5108	Leiognathidae	*Leiognathus ruconius*	62	92	1	127	282
Cluster5114	Engraulidae	*Encrasicholina pseudoheteroloba*	8	58	0	144	210
Cluster5226	Engraulidae	*Stolephorus andhraensis*	79	10	5	33	127
Cluster5162	Engraulidae	*Encrasicholina heteroloba*	6	16	2	31	55
Cluster4887	Dorosomatidae	*Hilsa kelee*	47	0	2	0	49
Cluster5015	Gobiidae	*Istigobius* sp1	7	21	1	11	40
Cluster4983	Apogonidae	*Ostorhinchus* sp.	13	8	13	5	39
Cluster3579	Engraulidae	*Engraulidae* sp.	2	20	1	11	34
Cluster4892	Nemipteridae	*Scolopsis vosmeri*	1	14	1	18	34
Cluster5183	Caesionidae	*Caesio cuning*	0	21	0	10	31
Cluster5090	Gobiidae	*Acentrogobius gracilis*	25	1	1	0	29
Cluster5232	Sciaenidae	*Pennahia anea*	18	5	0	6	29
Cluster5235	Gobiidae	*Cryptocentrus maudae*	2	21	0	4	28
Cluster5010	Gobiidae	*Arcygobius baliurus*	17	1	0	5	24
Cluster5064	Gobiidae	*Istigobius goldmanni*	1	20	0	3	24
Cluster4902	Lutjanidae	*Lutjanus carponotatus*	0	9	0	14	23
Cluster5151	Pseudochromidae	*Pseudochromis ransonneti*	0	18	2	2	22
Cluster4990	Butidae	*Butis koilomatodon*	19	1	0	1	21
Cluster5221	Gobiidae	*Cryptocentrus melanopus*	1	10	2	8	21

*Notes*: The final column represents the combined abundance across all sites in decreasing order. Summary of all clusters can be found in the Supporting information file 6.

The deep pugnose ponyfish *Leiognathus ruconius* Hamilton, 1822, was the most abundant taxon recovered at all sites, albeit only one specimen from Sisters (Table 4; Figure 1a). Gobiidae, a speciose family renowned for its cryptic diversity (Thacker & Roje, 2011; Tornabene et al., 2013), was the most species‐rich family in this study, comprising more than a quarter of all species sampled: 66 species (25.8% of all mOTUs). The second and third most speciose families were from Apogonidae Günther 1859 and Pomacentridae Bonaparte, 1831 with 20 species (7.8%) and 15 species (5.9%), respectively; each represented less than a third of the species from Gobiidae proportionally (Table 4; Supporting information file 7). From Gobiidae, the most abundant species was *Istigobius* sp1 (Table 4).

Of the fish larvae species sampled, majority would mature into adults that are not highly reef associated (Figure 3b; Supporting information file 7). Numerous species with adults that are only occasionally observed in reefs are also important as food fishes (e.g., threadfin breams Nemipteridae Regan, 1913 and snappers Lutjanidae) (Table 4; Figure 1; Supporting information file 7). Some fish larvae with juveniles and adults that are typically highly reef associated (e.g., *Plectorhinchus chrysotaenia* [Bleeker, 1855], *Chaetodontoplus mesoleucus* [Bloch, 1787]) were observed at sites (Tuas) further away from reefs (Supporting information file 7).

Two mOTUs initially matched to species with adults that are not found in the region of study—Cluster5237 matched to *Guentherus altivela* Osório, 1917, which are typically found in the Eastern Atlantic to Eastern Central Pacific (i.e., Portugal, Panama), and Singleton1771 had matched to *Scomberomorus plurilineatus* Fourmanoir, 1966, found in the Western Indian Ocean (i.e., Seychelles, Kenya) (Bussing & López, 1977; Collette et al., 2023; Murase et al., 2014) (Supporting information file 5). Scrutinizing the top NCBI *nt* hits for Cluster5237, we found that *G. altivela* (accession number: AP012957.1) was likely misidentified as all other ≥98% hits matched to *Psammogobius biocellatus* (Valenciennes, 1837), a more likely species found in this region. We accepted *P. biocellatus* as the final species identification of Cluster5237 due to the possibility that the GenBank entry for *G. altivela* was inaccurate (Supporting information files 4 and 7). Singleton1771 also matched ≥98% to another species more likely found in this region, *Scomberomorus guttatus* (Bloch & Schneider, 1801). We adopted the conservative approach for Singleton1771 by accepting the final species identification as *Scomberomorus* sp. on the account that both species identifications were possible (Supporting information files 4 and 7).

## 4 DISCUSSION

### 4.1 Reverse workflow for fish larvae identification

This study demonstrates the feasibility of a reverse workflow approach for sorting and identifying fish larvae with nanopore sequencing. Effective methods to sample fish larvae typically involve the use of light traps and plankton nets, as these can capture many specimens per standardized deployment. Consequently, our workflow that can effectively and rapidly identify specimens in line with the rate of collection is particularly desirable. Further, the identification of fish larvae using morphological characteristics typically discriminate specimens to familial levels, rarely achieving generic or specific levels (Azmir et al., 2017; Ko et al., 2013). This is especially true for closely related or speciose taxa with convergent morphometric counts or morphological traits. Here, we processed 3022 specimens, generated a total of 2746 barcodes after quality filtering (90.9% barcoding success), identified 2067 DNA barcodes (75.3% identification success), and delimited 256 mOTUs (146 genera, 52 families). This would have traditionally required more time and effort if specimens were to be morphologically sorted prior to sequencing. We selected several samples for morphological identification and found identifications accurate to family‐level hierarchies.

The technique we have adopted to extract DNA from a single eye of the larval specimens is advantageous because one side of the bilaterally symmetrical specimens can be preserved for measurements, examination, and photography (Appleyard et al., 2022; Motomura et al., 2013; Nonaka et al., 2021). For a small proportion of specimens with significant damages to one side, or those with missing eye(s), imaging occurs prior to tissue extraction. For specimens that were in poor condition beyond morphological identification, tissue was extracted without photography. We found the HotSHOT extraction to be straightforward and time‐efficient; the major bottleneck of the workflow was with extraction of tissue from specimens—unavoidable to preserve the morphology for post‐barcoding validation. Considering the large number of old specimens stored in fish collections worldwide, recent studies promoting the feasibility of extracting DNA from formalin‐fixed material may increase barcoding output, even though such an approach may require additional time and effort for de‐crosslinking of DNA (Appleyard et al., 2022; Hou et al., 2021). With an extensive database of images and voucher specimens accumulated over time, the need to preserve the morphology of well‐sampled species would also be reduced, accelerating the overall workflow.

### 4.2 Strengths and challenges of reference databases

The NCBI *nt* database is commonly used to obtain sequence matches for organism identification. We additionally used a curated local database to improve identification accuracy of our samples. Still, only 75.3% of barcodes were identifiable, suggesting existing gaps in sequence databases for fishes. The local database was able to identify barcodes not found in the NCBI *nt* database, demonstrating the importance of building, maintaining, and refining local databases (Delrieu‐Trottin et al., 2019; Ip et al., 2019; Oliveira et al., 2016).

Nevertheless, the use of two databases led to 18 conflicting identifications. Our overall approach was to be conservative: we accepted the matched identification of the highest taxonomic congruency from both databases when uncertain. Most disagreements were resolved easily (a single stepwise increase in taxonomic level, i.e., from species‐ to genus‐level identifications). The most substantial revision was for Cluster5177, which had originally matched identifications from different orders: *Sparus aurata* in NCBI *nt* database and *Omobranchus ferox* in the local database. Reasons for the acceptance of *O. ferox* as the final identification of Cluster5177 were threefold: (1) matches against full *S. aurata* mitochondrial sequences were low (85.6% at best), (2) phylogenetic analyses, which involved both mitochondrial (COI) and nuclear (ENC1, myh6, sreb2, tbr1) genes, placed GenBank entry MG210400 (99.4% match to Cluster5177) neatly within the *Omobranchus* clade (Gibbs et al., 2018), and (3) Cluster5177 matched 99.4% to *O. ferox* in the curated local database, with which we could match the barcode to voucher specimens. Cluster5237 was also originally misidentified in the NCBI *nt* database as *G. altivela* (99.2% match), a species highly unlikely to be found in this region; all other ≥98% matches were to *P. biocellatus*, a more probable species identification. We were unable to conclude which matched species was more accurate for Singleton1771 and took the conservative approach by accepting the genus *Scomberomorus* sp. instead of *S. plurilineatus* or *S. guttatus*. We are mindful to report observations of species that have not been sequenced before from this region for potential management implications of alien or invasive species. Although it is accessible and quick to conduct sequence matches to databases like NCBI, it is important to thoroughly verify the taxonomic identification of the matches, especially where voucher specimens are available (Wong et al., 2011).

It is also crucial to understand the target gene and the species delimitation threshold, which can be distinct between different taxon (e.g., COI not advised for most Anthozoa taxa due to slower rates of evolution) (France & Hoover, 2002; Hebert et al., 2003; Ratnasingham & Hebert, 2007; Huang et al., 2008; McFadden et al., 2011). The few disparate clusters with similar taxonomic identification suggest that relevant databases would need to be revised or updated for more accurate species identification.

Additionally, the use of other primers for alternative or multiple genes could provide greater taxonomic resolution, where species assignments are still unclear, for mOTUs that were delimited by a single gene. This is particularly useful for species placements using phylogenetic approaches.

### 4.3 Species richness and distribution

To fully understand species diversity across sites, it is important to achieve sampling completeness. Here, collections at Changi, Hantu, and Tuas appeared to have sufficiently sampled for species diversity. Sisters, the only marine protected area in Singapore, appeared to host the highest species diversity and would require greater sampling efforts to uncover more precise species diversity estimates.

Prevailing hydrodynamic patterns can influence the distribution of pelagic‐phase fish larvae. The hydrodynamics of waters surrounding Singapore are highly complex due to the culmination of parameters such as season, tides, and climatic effects (Kurniawan et al., 2015). Different tidal pressures of the Indian and Pacific Oceans cause the tidal range at the west of Singapore (i.e., Tuas) to be higher compared to the east (i.e., Changi) (Peng et al., 2023; Tay et al., 2016). The possibility of finding *S. plurilineatus* larvae in areas where adults are not typically found, such as shallow reefs or habitat types (e.g., deep benthic and neritic/oceanic, respectively), sheds light on the connectivity of ichthyoplankton across vast distances that are potentially driven by strong currents in the Singapore Strait.

### 4.4 Ontogenetic shifts and conservation

Fish larvae distribution is first and foremost influenced by strong hydrodynamics. High predation pressure at reefs could possibly lead to a tendency of aggregation of larvae that are soon to develop into juveniles at refugia sites close by reefs (Kimirei et al., 2013). The presence of fish larvae with reef‐associated adults at non‐reef sites (e.g., Tuas) suggests that there could be movement of larvae to other sites with different habitats (e.g., soft‐substrate habitats such as seagrass meadows and mudflats) before the return to reefs for adulthood. Larvae of reef‐associated taxa that were observed in non‐reef sites must journey back to coral reefs before metamorphosis, providing some evidence of ontogenetic shifts of reef‐associated fishes. Ontogenetic dietary changes and sexual maturation have been suggested as two key promoters of nursery to reef migrations in fishes (De La Morinière et al., 2003). However, high prey availability and predation pressure within reefs may lead to lower survival rates for smaller fishes, which could explain the benefit of taking refuge at adjacent habitats, resulting in high juvenile abundances in nearshore sites (such as Changi and Tuas) (Kimirei et al., 2013).

In Singapore, adult fish communities in coral reef and artificial seawall habitats show clear spatial variation (Taira et al., 2018). Although fish larvae may be found haphazardly across various habitats, reef settlement cues (e.g., live coral and high coral diversity) allow for juvenile or adult reef fishes to eventually settle and contribute to the clear spatial structuring of adult reef fish community, even within reef types (Chaput et al., 2019; Coker et al., 2014).

The sites sampled here are within an important channel for both fish larvae and vessels, connecting South China Sea in the Pacific with the Indian Ocean (Peng et al., 2023). Threadfin breams (e.g., white‐cheek monocle bream *Scolopsis vosmeri* [Bloch, 1792] and fork‐tailed threadfin bream *Nemipterus furcosus* [Valenciennes, 1830]) are important commercial fishes (Russell, 1990); the presence of their larvae in the Johor and Singapore Straits is indicative of their wide distribution, with records across the Indian and Pacific oceans (Hung et al., 2017). Ichthyoplankton of Lutjanidae snappers was sampled from non‐reef localities and habitat types (i.e., Hantu and Tuas). This reflects the distribution of their adults, which are occasionally observed in coral reefs as well as in non‐reef habitats such as seagrass and soft sediment (Sambrook et al., 2019). Lutjanidae comprises targeted species in commercial fisheries (e.g., trawls, bottom longlines, and trawls), extensively cultured or imported in aquaculture (e.g., Russell's snapper *Lutjanus russellii* [Bleeker, 1849] and John's snapper *Lutjanus johnii* [Bloch, 1792]), and even as popular game fish in recreational angling (e.g., Spanish flag snapper *Lutjanus carponotatus* [Richardson, 1842] and *L. johnii* [Bloch, 1792]) (Chou & Lee, 1997; Jaafar et al., 2012). The presence of commercially important fish in non‐reef sites suggests the importance of channels and non‐reef refugia sites, which should be considered together with reefs in conservation management.

## 5 CONCLUSION

Progress in DNA barcoding techniques has advanced the process of genetic taxonomic identification to be more accessible for bulk samples that are challenging to distinguish morphologically. Further developments in PCR technology (e.g., NextGenPCR thermocyclers) allow for full PCR amplification cycles to be completed under 30 min, accelerating a crucial step in DNA barcoding (Vasilita et al., 2024). The choice of sequencing platform is dependent on the specific research question, sample type, as well as time and budget limitations. Here, we focused on the use of nanopore sequencing technology with the MinION, which has been proven to “yield highly accurate barcodes that are 99.99% identical to Sanger barcodes” (Srivathsan et al., 2021; Srivathsan & Meier, 2024). Nanopore sequencing technology has also undergone expedited enhancements in flow cell and kit chemistry—the latest being R10.4.1 flow cell (FLO‐MIN114) and V14 kits—which boasts higher read quality and basecalling model accuracy above 99% (Vasilita et al., 2024; Zhao et al., 2023). To keep pace, related software for analysis of ONT data has been upgraded to suit higher read quality and effectively enable real‐time barcoding (Srivathsan et al., 2023). Today, the quality of DNA barcode data (including failure rates) produced by ONT is no longer a serious limitation; a major bottleneck of DNA sequencing is now the economics of obtaining and processing samples (Cuber et al., 2023). To this end, we demonstrated that the process of reverse workflow—the barcoding and sorting of all samples into mOTU clusters before identifying them (if needed)—for species identification is suitable for fish larvae, enabling more precise sampling of species diversity and better understanding of fish diversity and connectivity across the seascape.

## AUTHOR CONTRIBUTIONS

Wan Wen Rochelle Chan conceptualized the study, performed sample collection and laboratory work, conducted formal data analysis, and wrote the original draft. Jia Jin Marc Chang conceptualized the study, assisted in laboratory work, data analysis, and writing—review and editing. Charles Zhiming Tan performed sample collection and laboratory work. Jie Xin Ng and Matthew Hui‐Chieh Ng assisted in laboratory work. Zeehan Jaafar obtained funding, project administration, resources, and contributed to writing—review and editing. Danwei Huang provided funding, project administration, resources, supervision, conceptualized the study, and handled writing—review and editing.

## FUNDING INFORMATION

This research was supported by the National Parks Board Singapore (A‐0008413‐00‐00).

## CONFLICT OF INTEREST STATEMENT

The authors declare no competing interests.

## Supporting information


**File 1** Demultiplexing sheets for both runs and both primer pairs (FishF2 and FishR2, VF2 and FR1d) depicting unique 13‐bp forward (F) and reverse (R) custom tags for all samples sent for barcoding (3022 samples, 65 positive controls, 108 negative controls). The same combination of tags was used per sample for both primer pairs.

**File 2** Workflow for PCR amplification.

**File 3** Number of reads demultiplexed per run and primer pair. Median read depth is indicated in bold.

**File 4** Species identities of 2067 DNA barcodes (specimens) and database used for final identification (“conservative” was indicated for conflicting identities that were resolved via minor revisions, “revised” indicated that species identifications were corrected). Indicated in bold were clusters that merged at higher pair‐wise difference percentage (under cluster columns).

**File 5** Top hit for barcode clusters matched to NCBI *nt* database and the local database in order of decreasing percentage identity match (indicated in parentheses). Clusters with matches of ≥98% are indicated with “identifiable,” matches <98% were indicated as “without matches,” and matches for the positive controls were indicated as “control.” Clusters that matched ≥98% to both NCBI *nt* database and the local database to conflicting identifications are bolded for reference. Top hit for barcode clusters matched to BOLD database was also included for reference (percentage identity match was indicated in parentheses). BOLD process identity and additional information were also included in subsequent columns. Note that the following have updated taxonomic identifications: Cluster4104 *Oxyurichthys longicauda* is now accepted as *Oxyurichthys microlepis*; Cluster4530 *Moolgarda perusii* is now accepted as *Osteomugil perusii*; Cluster5232 *Pennahia anea* is now accepted as *Pennahia aneus*; Cluster5208 *Pomadasys hasta* is now accepted as *Pomadasys argenteus*; Cluster5244 *Repomucenus meridionalis* is now accepted as *Callionymus meridionalis*; Cluster 5210 *Repomucenus richardsonii* is now accepted as *Callionymus curvicornis*; Cluster 5112 *Yongeichthys nebulosus* is now accepted as *Acentrogobius nebulosus*.

**File 6** Samples removed for ecological analysis with respective reasoning stated in the final column.

**File 7** Sample abundance of all clusters and final identification per site. Typical habitat type information (reef, occasional reefs, non‐reef) for adults included. The final column represents the combined abundance across all sites in decreasing order.

## Data Availability

Fish larval vouchers (*n* = 3022) are currently still being processed by an ongoing project but will be deposited at the Zoological Reference Collection (ZRC) of the Lee Kong Chian Natural History Museum, Singapore. The *dorado*‐basecalled fastq files have been uploaded to NCBI Sequence Read Archive (SRA) under BioProject PRJNA1060255, and the DNA barcodes have been deposited at NCBI GenBank under accession numbers PP088104–PP090879.
