# Supplementary material for: Eyeing DNA barcoding for species identification of fish larvae
Source: J Fish Biol. 2024 Sep 3;105(6):1784–99. doi: 10.1111/jfb.15920 (PMC11650925; doi:10.1111/jfb.15920)
Supplement: Supplementary file 2 — File 2 Workflow for PCR amplification. [file JFB-105-1784-s002.pdf]

PCR Primer Tags [Example]

Each sample was placed in each well of numbered 96-well plates. For each sample, all four primers were used for PCR amplification (Figure 1). Forward primers had unique tags [**F tag**] per PCR plate (Table 1). Reverse primers had unique tags [**R tag**] per well-position (Table 2). Unique 13bp tags were obtained from Srivathsan et al. (2019; 2021).

FishF2_t1

5' – [**F tag**] TCGACTAATCATAAAGATATCGGCAC – 3'

FishR2_t1

5' – [**R tag**] ACTTCAGGGTGACCGAAGAATCAGAA – 3'

VF2_t1

5' – [**F tag**] TCAACCAACCACAAAGACATTGGCAC – 3'

FR1d_t1

5' – [**R tag**] CACCTCAGGGTGTCCGAARAAYCARAA – 3'

Figure 1: Forward and reverse primers with attached F and R tags respectively.

Table 1: Unique 13bp tags [**F Tag**] for forward primers used per PCR plate.

PCR Plate	F Tag
1	ATCCGGTCGGAGA
2	CTGAGGTGATCAG
3	AGTGTCCTGCTAG
4	ATAAGCAATTCGA
5	ATCTAACAGGACC
6	CATTATATAGCCA
7	CCTGATTACGTAA
8	CTAATACACACCG
9	TTATGACCGTTGC
10	TTCTCGGCCGCAT

Table 2: Unique 13bp [**R tag**] for reverse primers used for all PCR plates.

	1	2	3	4	5	6	7	8	9	10	11	12
**A**	ATCCGGTCGGAGA	TTATGACCGTTGC	TTGCGTCTCACGC	CTGTCGAGGCGAC	CAAGATCGGTACC	TTCGGCACAGGAG	TTCTTCTGGAACA	TACGAACTATAGC	CATGCACACCTCT	TCTAAGGAGTTAT	GGCTGTTACAACA	TGGTGGTCATTCA
**B**	CTGAGGTGATCAG	TTCTCGGCCGCAT	TTATTCGCACACT	GACTAATGAGGAA	CCAGCCTGACGGT	AGACTCGATTGAG	AGCAAGTGGCTCT	TACGGACCGCTCA	CGGAGAATATTAT	TCTATCTCTCGCT	GGTTCAATGACGC	TTACCAGAGCCTG
**C**	AGTGTCCTGCTAG	TTGGCGGTAAGAA	TGCACAGGCGGCA	TATCCGTAATCAT	CCGAAGCCGGTTC	ATCCTTCTGTTCT	TCAAGTCACTAAT	TGGACGCTAGATC	GAGCGAAGGTAGT	TGTGAAGTTGCCA	GGTTCCTGCACTT	TTCCGCGGCATAG
**D**	ATAAGCAATTCGA	ACTTGTGCACCTG	AACATGTGGTAAG	TCCTGGAGGATAA	CCGAGCAGCTGTT	CGTTACTTAGGTC	ATGAAGACTACTG	AGTCTTCACCTCC	GGCGACTGTTCCT	TTACCATTCCTCA	GTCCAACTTCAGT	ACAACCATTGGCA
**E**	ATCTAACAGGACC	CATGCCTTGATAC	ACGCGCTCTTATA	TCTACTGTTGTGC	CCTCCGCACAATC	CTGGTTGGCTATC	CACCGGTAGAACC	AGTGCTTATCCGT	GTCAGTTAACGAC	AGATTCTACACAA	GTCCAGGCCTTCT	AGCCTAGCCAACT
**F**	CATTATATAGCCA	CTGACGTTCGGTA	CAATGTTAATGGT	TGTATATTCAGCG	CTCTAACCTATTA	GCACCAACGATTG	CAGAACAACGCAT	ATCGCTGCCTGGC	TCCACAGATACAG	ATGCGATTAATTG	GTGACGCCGTATT	ATAATCGAGGCTT
**G**	CCTGATTACGTAA	GAGGAGAGACACA	CTAGGATATAGTT	ACATCAGTAGTTC	GCACCTTGACATA	TCTTATGCATAAC	CCTGCCGAGAACG	ATGCTTGCGACAG	TCGAGTTGTAACT	CCGTAGTGTTGAT	TGCAGCAACCAGC	CTGAGTCAGAAGT
**H**	CTAATACACACCG	GCCGGTCCAAGTG	CTGTAGGCGAGGA	AGCGCTGGACCAA	TAAGCGTGCTGAA	TTAGGTGTGGACC	GTGATGGAGCGGT	CAATCCTCAAGAG	TCGGTCTTAGACG	CCTGTACCATTGT	TGGACATGGCATT	GATAGGCGCCTTG
