# Supplementary figures and images for: Eyeing DNA barcoding for species identification of fish larvae
Source: J Fish Biol. 2024 Sep 3;105(6):1784–99. doi: 10.1111/jfb.15920 (PMC11650925; doi:10.1111/jfb.15920)

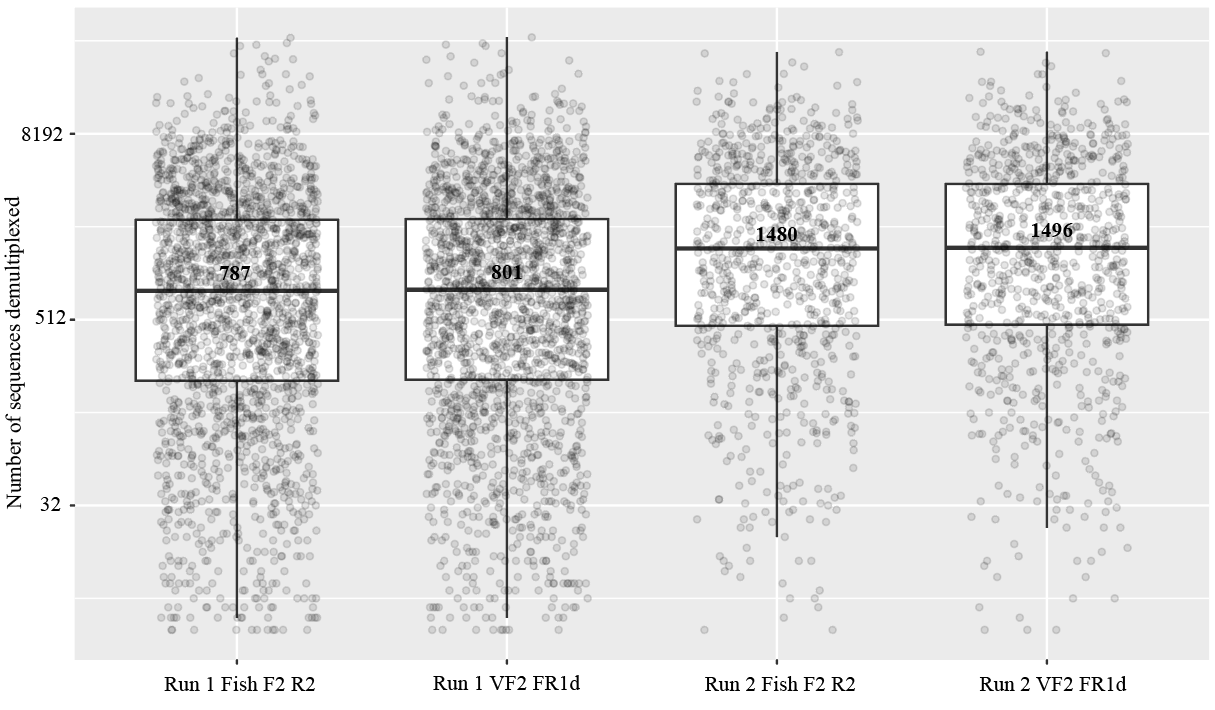

Supplement: Supplementary file 3 — File 3 Number of reads demultiplexed per run and primer pair. Median read depth is indicated in bold. [file JFB-105-1784-s007.png]
